# Primary and Secondary Stability of Subcrestal Morse-Taper Implants Placed Immediately into Extraction Sockets or into Grafted or Healed Ridges: A Retrospective Cohort Study

**DOI:** 10.3390/jcm15208184

**Published:** 2026-10-21

**Authors:** Jan Bublik, Alica Klieštiková, Kristína Frimelová, Petr Michl

**Affiliations:** 1 Bublik Dental Implant & Surgical Center, Wausau, WI 54401, USA; 2 Department of Oral and Maxillofacial Surgery, Faculty of Medicine and Dentistry, Palacký University Olomouc and University Hospital Olomouc, 779 00 Olomouc, Czech Republic; alicka.kliestikova@gmail.com (A.K.); kristina.frimelova@gmail.com (K.F.); petr.michl@fnol.cz (P.M.)

**Keywords:** dental implants, immediate implant placement, implant stability quotient, resonance frequency analysis, insertion torque, Morse taper, subcrestal placement, socket preservation

## Abstract

**Background/Objectives:** Immediate implant placement into fresh extraction sockets is often assumed to provide lower primary stability than placement into healed ridges, but comparative data for subcrestal Morse-taper (conical-connection) implants are lacking. This study compared insertion torque value (ITV) and implant stability quotient (ISQ) between two management protocols: immediate placement and delayed placement. **Methods:** This retrospective single-surgeon cohort included consecutive Argon K3 Pro implants placed in 2019–2022, grouped as immediate (IM), delayed into sites grafted within the preceding 12 months (DG) or delayed into healed ridges (HR). The protocols differed in more than timing (placement depth, grafting, flap design and healing management). ITV, ISQ at placement and ISQ at 3 months were compared with mixed-effects models accounting for multiple implants per patient. ISQ differences were tested against a post hoc ±5-unit equivalence margin; no equivalence test was applied to ITV. **Results:** Of 178 implants screened, 130 implants in 85 patients (52 women, 33 men) were analyzed: 56 IM, 30 DG and 44 HR. No difference was detected in ITV (delayed minus IM −1.4 Ncm, 95% CI −5.0 to 2.1), placement ISQ (IM 72.3 ± 7.8 vs. delayed 72.5 ± 9.0) or 3-month ISQ (78.8 ± 4.8 vs. 79.4 ± 5.6); in the primary analysis, the 90% confidence intervals of both ISQ differences lay within ±5 units, but the placement ISQ interval did not lie within ±3 units, and equivalence at placement was borderline in a size-adjusted sensitivity analysis. ISQ change was strongly related to starting ISQ (Spearman’s ρ = −0.77), largely through arithmetic coupling and regression to the mean, and baseline-adjusted 3-month ISQ did not differ between protocols (*p* = 0.46). All 19 immediate implants starting below ISQ 70 reached ISQ ≥ 70 by 3 months under the protocol used. In an exploratory analysis, ITV correlated with placement ISQ in healed ridges (ρ = 0.55) and more weakly in extraction sockets (ρ = 0.25); the difference between these correlations was inconsistent across methods. **Conclusions:** In this cohort, immediate and delayed protocols showed no detectable difference in insertion torque or ISQ, and ISQ differences were statistically within a post hoc ±5-unit margin. Whether torque is a weaker indicator of stability in extraction sockets is a hypothesis for prospective study.

## 1. Introduction

The timing of implant placement after tooth extraction remains a central decision in implant dentistry [1,2]. Placement into a healed ridge (delayed placement) is widely regarded as the reference approach, whereas immediate placement into a fresh extraction socket shortens treatment and may help preserve the alveolar soft- and hard-tissue architecture. A long-standing concern with immediate placement is primary stability: the socket is usually wider than the implant, so a coronal gap (jump space) remains between the implant and the socket walls, and anchorage depends largely on bone apical and lateral to the socket [3]. Systematic reviews report broadly comparable survival for immediate and delayed protocols, with a modest excess of early failures after immediate placement in some analyses [4,5].

Primary stability is assessed clinically in two ways. Insertion torque value (ITV) is the peak torque needed to seat the implant; it is recorded once and reflects the frictional resistance of the osteotomy. Resonance frequency analysis gives the implant stability quotient (ISQ, 1–100) a non-invasive, repeatable measure of the stiffness of the implant–bone complex that can be followed through healing. The two measures are related but not interchangeable, and their correlation varies widely between studies and clinical situations [6,7,8,9,10].

The evidence comparing the stability of immediate and delayed implants is mixed. A recent meta-analysis found ISQ at placement about 5.7 units lower in fresh sockets than in healed sites, with no significant difference in ITV, but rated the certainty of that evidence as very low [11]. Individual studies range from lower stability in immediate implants at placement and at follow-up [12,13], to no difference at placement or at loading [14,15,16,17], to lower primary but equal secondary stability [18,19]. Two further questions are rarely addressed. First, few studies separate delayed sites that were grafted at extraction (socket preservation) from native healed ridges, although both are common in practice and the grafted bone may behave differently [20,21,22]. Second, the implants studied have almost all been bone-level designs placed at the crest. For deeply subcrestal Morse-taper (conical-connection) implants, whose connection permits placement several millimeters below the crest [23,24,25], we found no comparison of immediate and delayed stability.

This study therefore compared ITV and ISQ at placement and ISQ at 3 months between immediate and delayed placement protocols (delayed placement into grafted or healed ridges), in a single-surgeon cohort of subcrestal Morse-taper implants. The protocols differed in more than timing, as described in Section 2.4, so the comparison is between management protocols and not between timing alone. The null hypothesis was that implant stability does not differ between the immediate and delayed protocols. Secondary aims were to describe the change in ISQ during healing and its dependence on the starting value, the relationship between ITV and ISQ by site type and the radiographic bone level at follow-up.

## 2. Materials and Methods

### 2.1. Study Design and Setting

This was a retrospective cohort study of consecutive patients treated with Morse-taper implants between 2019 and 2022 at a private implant-surgical practice (Bublik Dental Implant & Surgical Center, Wausau, WI, USA). It was conducted in accordance with the Declaration of Helsinki as an analysis of records generated during routine clinical care, and is reported according to the STROBE guidelines [26]. Clinical and radiographic data were abstracted from the practice’s records by two examiners (A.K. and K.F.) working as authorized remote users of the practice’s records system under its internal Health Insurance Portability and Accountability Act (HIPAA) confidentiality agreement; no records left the practice’s systems, this access has since been revoked and the analysis dataset was de-identified before analysis. All patients had given written informed consent for the treatment they received.

### 2.2. Participants

Adults aged 18 years or older, American Society of Anesthesiologists (ASA) physical status I or II, who received an Argon K3 Pro implant during the study period were eligible. Patients were not treated with implants if they smoked, had type 2 diabetes mellitus, a history of periodontal disease or acute infection at the implant site; for anterior immediate sites planned for a provisional restoration, a deep overbite (>4 mm) was an additional contraindication. For inclusion in the analysis, an implant required a recorded placement protocol, ITV and ISQ at placement and at 3 months. Implants lacking any of these were excluded. Placement timing was decided clinically at treatment planning and was not randomized; within the immediate group, provisionalization required an ITV of 25 Ncm or more.

### 2.3. Placement Groups

Implants were classified into three groups by placement protocol:

- IM (immediate): implant placed into a fresh extraction socket at the extraction visit.
- DG (delayed, grafted): implant placed into a site that had received a socket-preservation graft within the preceding 12 months. Most were implanted about 3 months after grafting (mean 4 months, range 3–9; three sites at 6, 9 and 9 months). Because nearly all intervals were close to 3–4 months, the interval was not analyzed as a covariate.
- HR (delayed, healed ridge): implant placed into a site that had healed by clot without grafting: the teeth had on average been missing for more than 12 months, patients reported no previous graft and no graft material was visible radiographically at implant placement.

DG and HR were pooled as the delayed group for the primary comparison and analyzed separately as secondary comparisons.

### 2.4. Implant System and Surgical Protocols

All implants were Argon K3 Pro (Argon Dental Vertriebs GmbH, Bingen am Rhein, Germany): tapered, grade 4 commercially pure titanium, acid-etched surface, with a Morse-taper (3° included angle 1.5° per side) implant–abutment connection. Diameters ranged from 3.0 to 5.0 mm on two restorative platforms (2 mm for 3.0–3.5 mm diameters, 3 mm for 4.0 mm and above). All implants were placed by one surgeon (J.B.), and the definitive restorations were made by different dental laboratories.

Delayed placement (DG and HR). Implants were placed with an open flap into a prepared osteotomy, 1–2 mm below the mid-buccal bone crest and submerged for primary-intention healing. No graft was placed at implantation in either delayed group. Socket-preservation grafts in DG sites had used mineralized cancellous freeze-dried bone allograft (FDBA; OSSIF-i, Surgical Esthetics, Duluth, GA, USA; particle size 0.25–1.0 mm).

Immediate placement (IM). After an atraumatic extraction, with no flap raised and thorough debridement of the socket, the implant was anchored in native bone at least 2–3 mm beyond the apex of the socket where anatomy permitted and positioned 3–4 mm below the mid-buccal crest (platform about 4–5 mm below the mid-buccal free gingival margin). The jump space around and coronal to the platform was filled with the same FDBA hydrated (about 2 mL) with sterile saline containing 50 mg/ml cefazolin, an empirical measure based on implant-pharmacology teaching [27] for which we know of no controlled evidence and covered with a resorbable collagen dressing (collagen tape; Surgical Esthetics). Implants with ITV below 25 Ncm were submerged beneath at least 2 mm of allograft; implants with ITV of 25 Ncm or more received a custom tissue former or a screw-retained poly(methyl methacrylate) (PMMA) provisional crown kept out of occlusal contact, which was checked with articulating paper in maximal intercuspation and in protrusive and lateral excursions.

These two protocols therefore differed in placement depth (3–4 mm vs. 1–2 mm below the mid-buccal crest), flap design (no flap for immediate implants, open flap for delayed implants), apical engagement beyond the socket, grafting of the jump space and healing management (submerged or provisionalized vs. submerged). Placement depth, grafting, and provisionalization are defined by the protocol and are therefore collinear with group; they could not be separated statistically.

Healing period. Delayed implants were uncovered at second-stage surgery 12 weeks after placement. Immediate implants were reviewed at a corresponding 12-week visit.

### 2.5. Outcome Measures

Insertion torque value (ITV, Ncm) was read from the calibrated surgical motor at final seating (iChiropro, Bien-Air Dental SA, Bienne, Switzerland).

Implant stability quotient (ISQ) was measured with a Penguin RFA instrument and MulTipeg transducers (Integration Diagnostics Sweden AB, Gothenburg, Sweden) in four directions. The highest of the four values was recorded at placement and the lowest recorded value at the 3-month visit, with the same transducer type and operator at both visits; the individual directional values were not retained. The convention was chosen for a clinical reason. During healing, the clinician’s main question is whether stability has decreased, and recording the highest value at placement and the lowest at 3 months answers it conservatively: a 3-month reading at or above the placement reading means that stability did not fall in any measured direction. Averaging the four directions at each visit would describe mean stability more precisely but could conceal a decrease in one direction, and it was not practical chairside. Inter-examiner and test–retest reliability of ISQ were not assessed. ISQ was measured at placement and at the 12-week visit (second-stage surgery for delayed implants); for immediate implants with a provisional crown, the crown was removed to attach the MulTipeg. ISQ change was defined as 3-month ISQ minus placement ISQ.

Radiographic bone level (descriptive). For implants with a follow-up radiograph taken at least 3 months after placement, peri-implant bone level was measured on the most recent available film: bitewings for posterior and periapicals for anterior implants, taken with a Carestream digital sensor (Carestream Dental, Atlanta, GA, USA) and a film holder, and measured in ORYX (ORYX Dental Software AI plan). Each image was calibrated by fitting the on-screen ruler to the known implant dimensions. The vertical distance from the implant platform to the most coronal bone-to-implant contact was measured mesially and distally, with negative values denoting bone coronal to the platform and positive values bone apical to it. Each implant was measured once, by one of the two examiners. Reliability was assessed post hoc in a subsample: one author (P.M.), blinded to the original values, re-read 20 implants (40 sites); the absolute values agreed with the original readings (intraclass correlation coefficient 0.93), and 37 of 40 sites were within 0.5 mm, although the sign convention differed at some sites. Because the placement radiographs were not measured, these values describe bone level at follow-up, not bone change.

Other variables were jaw and region (from the tooth number) and implant diameter and length, retrieved from the practice’s implant inventory log by patient, tooth and surgery date.

### 2.6. Data Verification

One 3-month ISQ value that was blank in the abstraction sheet was retrieved from the clinical chart (ISQ 82). Implants whose ITV had not been recorded were excluded rather than imputed.

### 2.7. Statistical Analysis

Continuous variables are reported as mean ± standard deviation (SD), median and range. Because many patients received more than one implant, all inferential comparisons accounted for clustering within patients. The primary between-group estimates came from linear mixed-effects models (statsmodels MixedLM, restricted maximum likelihood) with a random intercept for each patient (variance-components structure, i.e., exchangeable correlation among a patient’s implants) and placement protocol (delayed vs. immediate) as the fixed effect. Models were fitted without and with adjustment for implant region (maxillary anterior, maxillary posterior, mandibular anterior, mandibular posterior), because immediate placement was preferred in the maxillary anterior region. Placement depth, grafting and provisionalization were not entered because they are determined by the protocol. In sensitivity analyses, models were restricted to posterior sites, restricted to the 88 implants with recorded diameter and length and adjusted for both, and repeated in all screened implants with the measure available (available-case analysis). Results are given as the delayed-minus-immediate difference with a 95% confidence interval (CI). Residuals were mildly left-skewed (skewness −0.7 to −0.9) with intraclass correlations of 0.08 for ITV and 0.19–0.28 for ISQ; no transformation was applied, and patient-level cluster bootstrap intervals (5000 resamples of patients) were computed as a robustness check. Three-group comparisons used likelihood-ratio tests of mixed models fitted by maximum likelihood, and within-group ISQ change was estimated with intercept-only mixed models. ISQ change was also analyzed as 3-month ISQ adjusted for placement ISQ (analysis of covariance within the mixed model) [28].

To judge whether any ISQ difference was clinically relevant, two one-sided tests (TOST) of equivalence were applied [29], using the 90% CI of the mixed-model difference and a margin of ±5 ISQ units, and exact TOST *p* values are reported. The margin was set below the 5.7-unit pooled difference reported in a recent meta-analysis [11] and corresponds to 0.6 SD of placement ISQ and 0.95 SD of 3-month ISQ in this cohort. It was not prespecified in a protocol; it was chosen during analysis, so the equivalence results are not confirmatory. To show how far the conclusion depends on the margin, TOST was repeated with ±4 and ±3 units, and the smallest margin supported by each interval is given. No equivalence margin was defined for ITV, and ITV is described only by the difference and its CI.

The association between ITV and ISQ was assessed as an exploratory analysis with Spearman’s ρ by group; 95% CIs were obtained by resampling patients (cluster bootstrap), and the HR and IM correlations were compared with Fisher’s z-test (which treats implants as independent) and with a cluster bootstrap of the difference. Effect modification was also tested with a mixed model containing ITV (per 10 Ncm), placement group and their interaction. No adjustment was made for multiple comparisons in these exploratory analyses. Because the change score contains the baseline reading, the relation between starting ISQ and ISQ change was examined with Pearson’s and Oldham’s correlations and interpreted descriptively only; the Pearson correlation is determined by the variances of the two readings and their correlation [30]. Recovery of implants with low placement stability (ISQ < 70) was described by group. Two-sided *p* < 0.05 was considered significant. Analyses were performed in Python 3.14 with SciPy 1.18, statsmodels 0.15 and pandas 3.0; the analysis code is provided as Supplementary Materials (File S2).

### 2.8. Use of Generative Artificial Intelligence

Claude Opus 5.5 (Anthropic, San Francisco, CA, USA) was used to write the Python code for the statistical analyses and figures, to check the abstracted dataset for internal inconsistencies (each flagged item was verified against the clinical records by the authors) and to assist with drafting and language editing of the manuscript. The authors reviewed all code, outputs and text and take full responsibility for the content.

## 3. Results

### 3.1. Study Cohort

**Figure 1:**
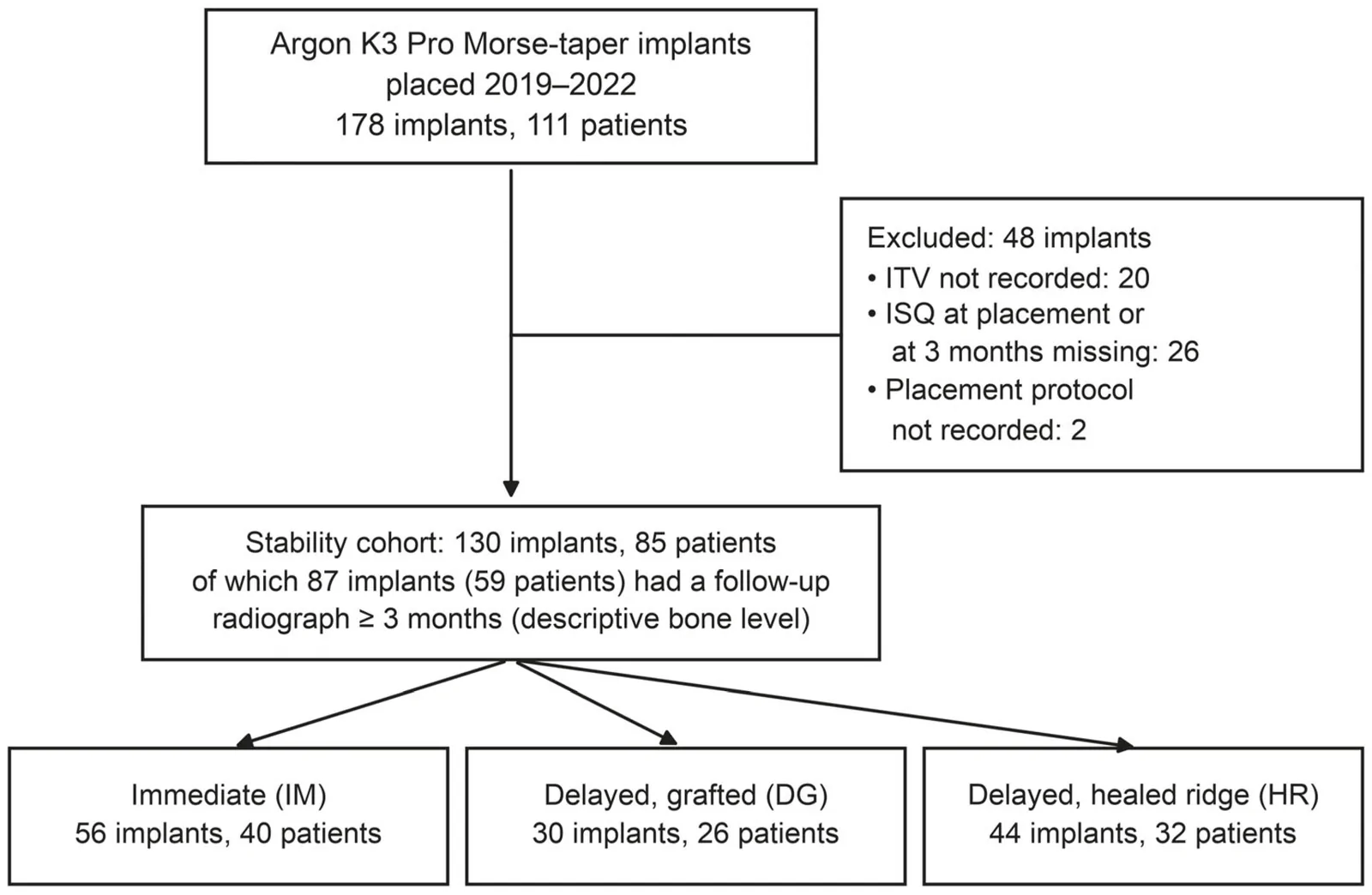
Flow of implants and patients through the study. ITV, insertion torque value; ISQ, implant stability quotient.

Of 178 implants placed in 111 patients during the study period, 48 were excluded: ITV not recorded (20), ISQ at placement or at 3 months missing (26) and placement protocol not recorded (2). Among the 46 excluded implants with a known protocol, 13 of 69 immediate (19%), 11 of 41 DG (27%) and 22 of 66 HR implants (33%) were excluded (χ^2^ *p* = 0.16). Excluded and included implants had a similar anatomical distribution (maxillary anterior 22% in both; *p* = 0.26) and, where recorded, similar placement ISQ (73.4 ± 7.8, *n* = 40, vs. 72.4 ± 8.5) and ITV (29.4 ± 5.3 Ncm, *n* = 18, vs. 30.7 ± 9.3). The analysis cohort comprised 130 implants in 85 patients: 56 IM (40 patients), 30 DG (26 patients) and 44 HR (32 patients) (Figure 1). The patients comprised 52 women and 33 men aged 21–82 years (mean 54 ± 12 years). Seventy-eight implants were in the maxilla and 52 in the mandible. Patients received 1 to 6 implants (63 patients had one implant, 22 had two or more, accounting for 67 of the 130 implants), and 13 patients contributed implants to more than one placement group, 11 of them to both the IM and a delayed group. Age and sex are reported for the cohort as a whole, because they were not abstracted by placement group. Maxillary anterior sites accounted for 38% of immediate implants but only 7% of DG and 14% of HR implants (Table 1). Diameter and length were recorded for 88 of the 130 implants (IM 64%, DG 80%, HR 64%); the median length was 9 mm in all three groups and the median diameter 4.25–4.5 mm.

### 3.2. Stability at Placement

No difference in ITV or placement ISQ was detected between protocols (Table 2). Mean ITV was 31.2 ± 8.9 Ncm for immediate and 30.2 ± 9.6 Ncm for delayed implants (mixed-model difference −1.4 Ncm, 95% CI −5.0 to 2.1; *p* = 0.42); the interval excludes differences larger than about 5 Ncm in either direction, but no equivalence margin was set for ITV and equivalence was not tested. Placement ISQ was 72.3 ± 7.8 and 72.5 ± 9.0 (difference −0.5, 95% CI −3.6 to 2.6; *p* = 0.76). The 90% CI of the ISQ difference (−3.1 to 2.1) lay within the ±5-unit margin (TOST *p* = 0.002), and so did the region-adjusted interval (−3.7 to 1.4; *p* = 0.007). The interval was not within ±3 units (TOST *p* = 0.055 unadjusted, 0.115 region-adjusted), so the data support a margin of about 3–4 units at placement but not narrower. The three groups did not differ in ITV (likelihood-ratio *p* = 0.59) or placement ISQ (*p* = 0.70).

### 3.3. Stability at 3 Months and Change During Healing

**Figure 2:**
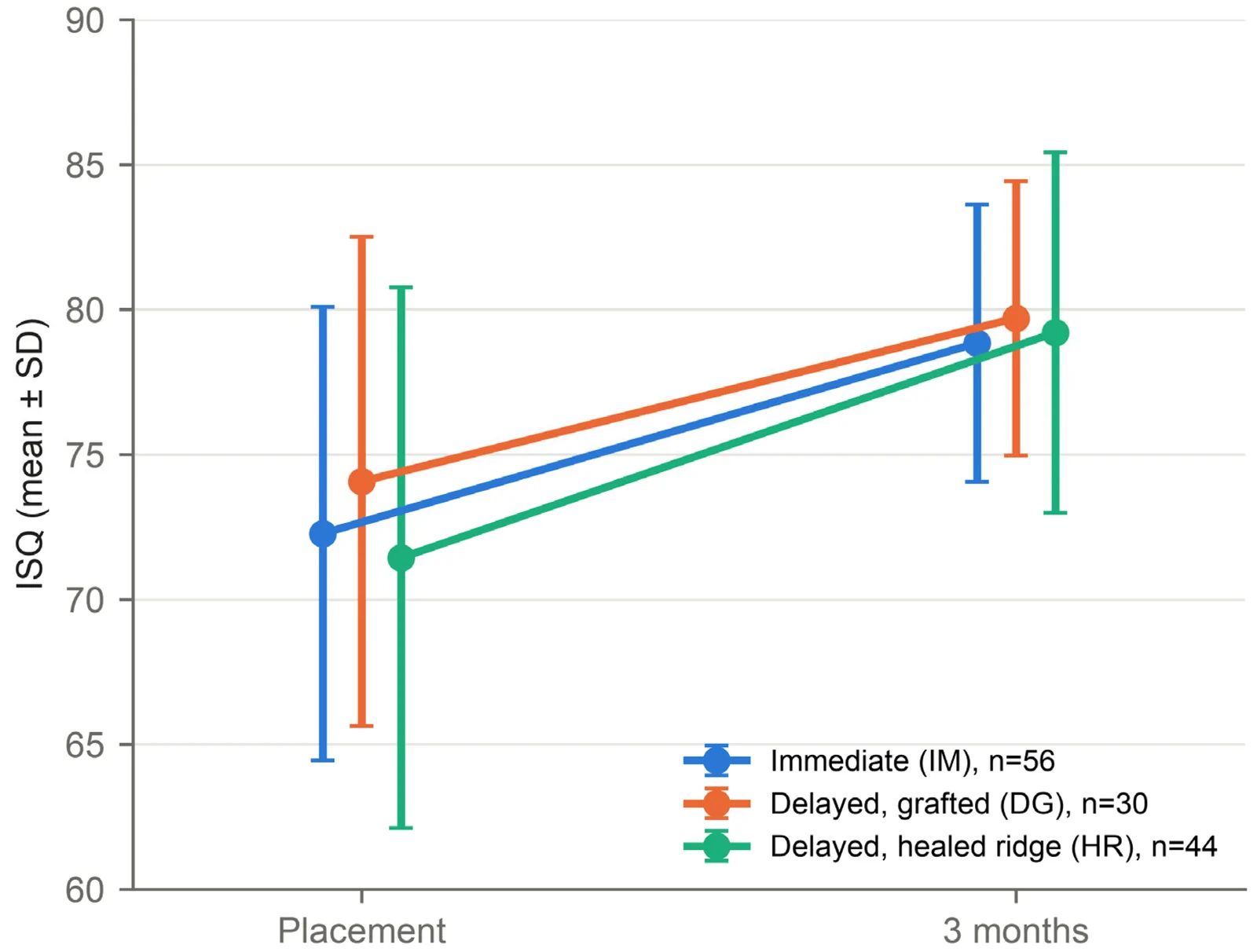
ISQ at placement and at 3 months by placement group (mean ± SD).

ISQ rose from placement to 3 months in every group (mixed-model change: IM +6.5, 95% CI 4.4 to 8.6; DG +5.5, 2.6 to 8.4; HR +7.2, 4.7 to 9.7; all *p* < 0.001; Figure 2). At 3 months, ISQ was 78.8 ± 4.8 for immediate and 79.4 ± 5.6 for delayed implants (mixed-model difference 0.5, 95% CI −1.3 to 2.4; *p* = 0.57). The 90% CI (−1.0 to 2.1) lay within the ±5 margin (TOST *p* < 0.001) and within ±3 units (*p* = 0.004); adjustment for region did not change this (difference 0.4, 90% CI −1.1 to 1.9).

**Figure 3:**
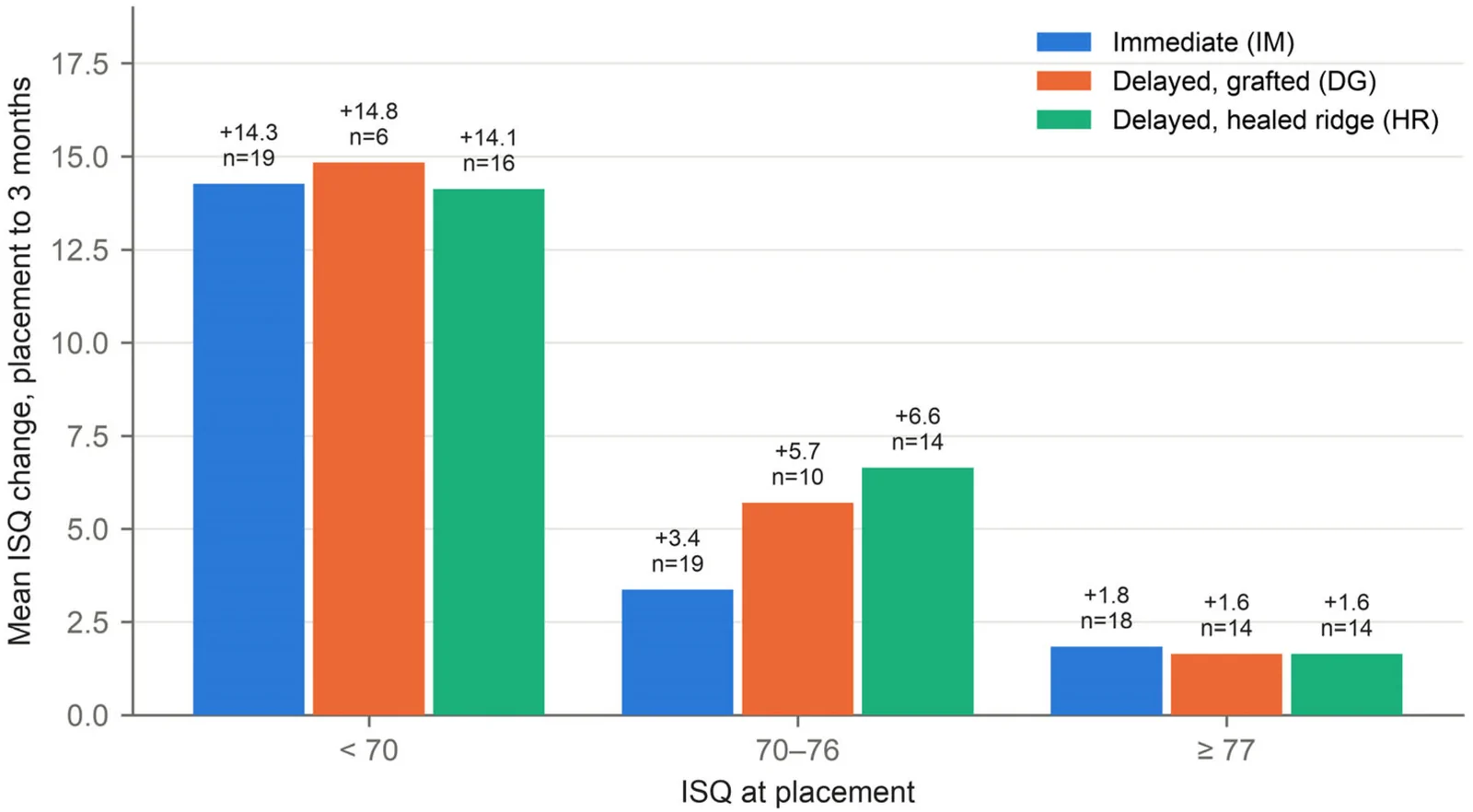
Mean ISQ change from placement to 3 months, by placement ISQ band and placement group. Numbers above bars give the mean change and number of implants. Because change scores contain the baseline reading, the decline of the gain with increasing starting ISQ partly reflects regression to the mean and mathematical coupling and should not be read as a purely biological effect.

The size of the ISQ change depended strongly on the starting value (Spearman ρ = −0.77, *p* < 0.001): implants that started low gained most, and implants that started at 77 or above changed little (Figure 3). This relation is largely arithmetic. The change score contains the baseline reading, and the Pearson correlation between placement ISQ and ISQ change (−0.79) follows directly from the SDs of the two readings (8.5 and 5.3) and their correlation (0.51); it is not independent evidence of a biological effect. Descriptively, the between-implant SD fell from 8.5 at placement to 5.3 at 3 months, and 85% of implants lay at 75 or above at 3 months. Whether that narrowing reflects healing, regression to the mean, a ceiling of the ISQ scale or the different recording conventions at the two visits (Section 2.5) cannot be determined from two time points. With placement ISQ as a covariate, 3-month ISQ did not differ between protocols (difference 0.6, 95% CI −1.0 to 2.2; *p* = 0.46; region-adjusted 0.7, 95% CI −0.9 to 2.3; *p* = 0.40), nor between IM and either delayed subgroup (analysis of covariance: DG *p* = 0.78, HR *p* = 0.50). The mixed-model estimates are summarized in Table 3.

No sensitivity analysis detected a difference between protocols (Table 3), but statistically demonstrated equivalence was less consistent, particularly at placement. In posterior sites only (IM 34, DG 28, HR 35), where the regional imbalance is removed, the placement ISQ difference was −1.6 (95% CI −5.0 to 1.7; 90% CI −4.4 to 1.2; TOST P at ±5 = 0.024), and the 3-month difference 0.1 (95% CI −2.0 to 2.2). In the 88 implants with recorded size, adjustment for diameter, length and region gave a placement ISQ difference of −1.8 (90% CI −5.0 to 1.4; TOST *p* = 0.050), so equivalence at ±5 was borderline at placement in this subset and equivalence at placement was not shown at ±3 units in any analysis, or at ±4 units in the posterior-only and size-adjusted analyses, and a 3-month difference of 0.4 (95% CI −1.7 to 2.6). Using every screened implant with the measure available (available-case analysis: 170 for placement ISQ, 150 for 3-month ISQ, 148 for ITV) also detected no difference (ISQ differences −0.5 and 0.4; ITV difference −2.1 Ncm, 95% CI −5.2 to 1.0). Patient-level cluster bootstrap intervals were similar to the model intervals (placement ISQ 95% CI −2.7 to 3.4; 3-month ISQ −1.0 to 2.2; ITV −4.5 to 2.5).

### 3.4. Implants with Low Placement Stability

Forty-one of the 130 implants (32%) had a placement ISQ below 70: 19 of 56 immediate implants (34%), 6 of 30 DG implants and 16 of 44 HR implants. By 3 months, 38 of the 41 had reached ISQ 70 or more, including all 19 immediate implants (DG 5/6, HR 14/16); the proportion recovering did not differ between the 19 immediate and the 22 delayed implants below 70 (19/19 vs. 19/22; Fisher *p* = 0.24). Overall, 6 of 130 implants (4.6%) were below ISQ 70 at 3 months (IM 2, DG 1, HR 3) and 110 (85%) were at 75 or above. By the protocol rule, 4 of the 19 low-ISQ immediate implants had ITV below 25 Ncm and were submerged (placement ISQ 61.2, 3-month ISQ 79.5, minimum 76) and 15 had ITV of 25 Ncm or more and received a tissue former or provisional crown out of occlusion (63.6 and 76.8, minimum 71) (management was inferred from the ITV rule of the protocol and was not abstracted per implant). The 23 implants with ITV below 25 Ncm, which were submerged, started at a mean ISQ of 65.7 ± 9.6 and reached 77.0 ± 7.2 at 3 months, compared with 73.8 ± 7.5 and 79.6 ± 4.7 for implants seated at 25 Ncm or more. Among immediate implants alone, 3-month ISQ was 78.3 for the 7 with ITV below 25 Ncm and 78.9 for the 49 with 25 Ncm or more.

### 3.5. Relationship Between Insertion Torque and ISQ (Exploratory)

Across the cohort, ITV correlated moderately with placement ISQ (ρ = 0.34, 95% CI 0.16 to 0.50) and weakly with 3-month ISQ (ρ = 0.22, 95% CI 0.07 to 0.37). By group (Table 4), the association was strongest in healed ridges (placement ρ = 0.55, 95% CI 0.25 to 0.78; 3 months ρ = 0.41, 95% CI 0.12 to 0.68) and weaker in fresh sockets (placement ρ = 0.25, 95% CI −0.04 to 0.50; 3 months ρ = 0.07, 95% CI −0.22 to 0.36) and grafted ridges. Evidence that the association differs between protocols was inconsistent. The difference between the HR and IM placement correlations did not reach significance by Fisher’s z-test (z = 1.74, *p* = 0.082) or by a patient-level cluster bootstrap (difference 0.30, 95% CI −0.09 to 0.67; *p* = 0.13). In a mixed model with an ITV-by-group interaction, placement ISQ rose by 5.9 units per 10 Ncm in healed ridges (95% CI 3.5 to 8.3) and 2.1 units in fresh sockets (95% CI −0.05 to 4.2); the interaction was significant for IM vs. HR (difference 3.9 units per 10 Ncm, 95% CI 0.8 to 6.9; *p* = 0.015) and across the three groups (*p* = 0.044), but not for 3-month ISQ (*p* = 0.23 for IM vs. HR; *p* = 0.33 across three groups). These analyses were not adjusted for multiplicity.

### 3.6. Radiographic Bone Level at Follow-Up (Descriptive)

Eighty-seven implants in 59 patients (33 immediate, 54 delayed) had a follow-up radiograph at least 3 months after placement; 43 did not (IM 23 of 56, DG 6 of 30, HR 14 of 44), because the restoring dentists did not send the later films despite repeated requests. Follow-up was a median of 10 months overall (range 3–41; IM median 9, interquartile range 4–19; DG 19.5, 8.5–27; HR 9, 6–18), and 65 implants had at least 6 and 41 at least 12 months of follow-up. At follow-up, mean bone level lay coronal to the implant platform in both groups: mesially −1.2 ± 1.5 mm for immediate and −0.8 ± 1.5 mm for delayed implants, and distally −1.1 ± 1.6 mm and −0.6 ± 1.6 mm. Because these data are descriptive, no between-group test is reported. One delayed implant had no measurable mesial value (mesial *n* = 53, distal *n* = 54). Bone lay apical to the platform at 8 of 33 immediate and 19 of 53 delayed implants mesially, and at 8 of 33 and 16 of 54 distally. Because immediate implants were placed about 2 mm deeper than delayed implants by protocol, the placement radiographs were not measured and follow-up differed between groups, these values are descriptive only; they cannot be read as bone loss or gain, and they do not support conclusions about comparative bone preservation or peri-implant tissue stability.

### 3.7. Implant Failure

One implant in the 178 screened failed during the study period (0.6%): a delayed healed-ridge implant in the anterior mandible that did not osseointegrate and was removed at second-stage surgery. It had no 3-month ISQ because it was removed and could not be measured, so it is not among the 130 analyzed implants; its exclusion was therefore related to the outcome. No other failure was recorded in the practice’s records for the 177 other screened implants, including the 47 excluded for missing data, and no immediate implant was recorded as lost. Failure status was obtained from the practice’s records and was not ascertained systematically over a defined follow-up period; failures after patients returned to their restoring dentists may not have been recorded, so these figures are the proportion of implants with a documented failure (1 of 178, 0.6%), not a survival rate. In a worst-case sensitivity analysis, the failed implant was added with an imputed 3-month ISQ of 61 (the lowest observed), 40 or 30; the delayed-minus-immediate difference at 3 months remained between −0.1 and 0.3 (90% CI −2.1 to 1.9), within ±5 and within ±3 units (TOST *p* ≤ 0.007).

## 4. Discussion

In this cohort of 130 subcrestal Morse-taper implants, we detected no difference in stability between an immediate placement protocol and delayed placement into grafted or healed ridges. Insertion torque, ISQ at placement and ISQ at 3 months did not differ, and for both ISQ time points, the confidence interval of the difference lay within a post hoc margin of ±5 units. That is a statement about the specific protocols used here, which differed in placement depth, grafting and healing management as well as timing, and not about timing alone. The margin was chosen during the analysis, and the data would support a margin of about 3–4 units at placement and about 2 units at 3 months, but not narrower ones; the conclusion is therefore one of no detected, and probably small, difference, not of proven equivalence. At placement, equivalence even at ±5 units was borderline in the size- and region-adjusted subset (TOST *p* = 0.050), so the placement result in particular should be read as absence of a detected difference. The ±5 margin is a judgment: it is smaller than the pooled placement difference of 5.7 units reported for fresh versus healed sites [11] and about 0.6 SD of placement ISQ in this cohort, but whether a 3–5-unit difference matters clinically is not established, because ISQ is a surrogate of implant–bone stiffness and no cut-off has been validated against survival or loading readiness [31]. For ITV, no equivalence claim is made: the interval excludes differences larger than about 5 Ncm, but a margin was neither defined nor tested.

The absence of a detected stability deficit for immediate placement contrasts with the pooled estimate of a recent meta-analysis, which found placement ISQ about 5.7 units lower in fresh sockets [11], and with single-center series in which immediate implants remained less stable at the 2–3-month review [12,13]. It agrees with trials that found no difference at placement or at loading [14,15,16,17]. The same meta-analysis noted that the deficit disappeared when wider implants were used in sockets [11], and the protocol here was designed to maximize anchorage in native bone: implants were engaged at least 2–3 mm beyond the socket apex and placed deeply, so a long segment of the implant was seated in bone below the socket. This may explain why placement ISQ in the immediate group matched that of healed ridges, but the present design cannot separate the contributions of implant geometry, apical engagement, placement depth, jump-space grafting and provisionalization, because these are built into the protocols. ISQ reflects the stiffness of the implant–bone complex [31]; the transducer is attached through the same internal connection in every group, so any effect of the connection design is shared by all groups and is not expected to differ between protocols.

The course of ISQ during healing is best described cautiously. Implants below ISQ 70 gained about 14 units, whereas those at 77 or above gained fewer than 2, and the gain within each band of placement ISQ was similar for immediate, grafted and healed sites (Figure 3). The strong negative correlation between starting ISQ and ISQ change is, however, partly expected from the mathematics of change scores and from regression to the mean, because the baseline reading appears on both sides of the relation [30]; the observed correlation is fully determined by the variances of the two readings and their correlation, so it cannot by itself show biological convergence. Between-implant variability fell from 8.5 to 5.3 units, and most readings lay in a narrow range around 79–80 at 3 months, but this narrowing may reflect regression to the mean, a ceiling of the scale or the recording convention as well as healing, and is reported descriptively only. The size of the change should also be read with the recording convention in mind: the highest directional value was recorded at placement and the lowest at 3 months, so the two readings are differently defined summaries and the recorded change is not a change in an identically defined measurement. Because the lowest value at 3 months cannot exceed, and the highest value at placement cannot fall below, the value in any single direction, the recorded change is at most equal to the change in any one direction, but its true magnitude cannot be recovered from the retained data. Comparing protocols with placement ISQ as a covariate avoids the coupling problem and showed no difference at 3 months [28]. The pattern resembles the healing trajectory described for low-torque implants [32] and studies in which immediate implants that started lower gained more [18].

Every immediate implant with a placement ISQ below 70 (*n* = 19) reached 70 or more by 3 months, as did 19 of 22 delayed implants (*p* = 0.24). Because placement ISQ was the highest and 3-month ISQ the lowest directional value, these implants were below 70 in every direction at placement and at or above 70 in every recorded direction at 3 months; the size of their gain, however, is subject to the measurement and regression-to-the-mean caveats above. This is an observation of what happened under the management protocol of this practice: four of the 19 had ITV below 25 Ncm and were submerged, and 15 received a tissue former or a provisional crown kept out of occlusion. Whether the same recovery would follow other loading strategies, or whether low initial stability should instead lead the clinician to reconsider immediate placement, cannot be answered by this retrospective cohort. Published ISQ cut-offs differ: values of 54 or 60 and above have been used for immediate loading and to predict integration [33,34], about 60.5 at 8 weeks predicted failure of immediately restored implants [35], and 70 has been used as the threshold for early loading [36]. ISQ 70 is also the value used in the surgeon’s own protocol, but it is a surrogate threshold: reaching 70 shows that the stiffness measure improved, not that osseointegration was complete, that the implant would survive, or that it was ready for functional loading [31]. The one failed implant in the screened cohort, a delayed healed-ridge implant with no 3-month ISQ, was necessarily absent from the stability analysis, so the data say nothing about whether low stability predicts failure. One documented failure among 178 screened implants, none of them immediate, is compatible with but does not demonstrate the safety of immediate placement, and it is not a survival estimate, because failure status was not ascertained over a defined follow-up period for every implant.

The relationship between insertion torque and ISQ may depend on the site, and we regard it as a hypothesis. In healed ridges, where the implant is surrounded by bone along its whole length, torque and ISQ moved together (ρ = 0.55, 95% CI 0.25 to 0.78). In fresh sockets the correlation was weaker (ρ = 0.25, 95% CI −0.04 to 0.50) and absent by 3 months (ρ = 0.07). The evidence that these correlations differ is inconsistent: Fisher’s z-test (*p* = 0.082) and a cluster bootstrap (*p* = 0.13) were not significant, whereas a linear interaction model was (*p* = 0.015 for IM vs. HR at placement), and no analysis was adjusted for multiplicity or for other covariates. A possible explanation is geometric: torque in a socket is generated mainly where the implant engages bone apically and at the socket walls, whereas ISQ reflects lateral stiffness, which is reduced where the coronal part of the implant faces the grafted jump space. Our finding agrees with a study of immediate implants that found no torque–ISQ correlation [9] and with a systematic review reporting no significant overall correlation [6], but it is the opposite of a randomized trial that found a stronger correlation in post-extraction than in healed sites [14]. If confirmed in prospective data, torque alone might be a less reliable guide to stability in immediate sites, but this study does not establish that.

Delayed implants in grafted sites behaved like those in native healed ridges: placement ISQ, 3-month ISQ and ISQ change were similar in DG and HR. This agrees with reports that implants in preserved sockets reach stability comparable to native bone [21,22] and that grafted bone given enough time to mature approaches or exceeds native bone in ISQ [20]. Most DG sites were implanted 3 months after grafting, an interval shorter than in some of those studies, and because nearly all intervals were 3–4 months the effect of the interval could not be examined.

Radiographic bone level was reported only descriptively. On the latest available film, mean bone level lay about 1 mm coronal to the platform in both groups, and somewhat more coronal in immediate sites. The placement radiographs were not measured, immediate implants were placed about 2 mm deeper by protocol, follow-up length differed between groups and 43 implants had no usable film, so the difference probably reflects placement depth and is not evidence of bone gain or of better bone preservation; subcrestal positioning is itself associated with more coronal bone around the platform [25,37]. These data show that bone remained at or above the platform at most implants over a median of 10 months, but they cannot show whether marginal bone was lost or gained.

This study has limitations. It is retrospective and from a single practice, with one surgeon and one implant system, which keeps the protocol consistent but limits generalization. Protocol was chosen clinically, not randomly, and the protocols differ in placement depth, grafting, flap design and healing management, so the groups differ in more than timing; these factors are collinear with group and could not be adjusted for. Immediate placement was preferred in the maxillary anterior region (38% of immediate vs. 7% and 14% of DG and HR implants); adjustment for region and a posterior-only analysis did not change the estimates, but residual confounding by bone density, implant dimensions (recorded for 88 of 130 implants, with similar results when adjusted) and case selection cannot be excluded, and age and sex could not be compared between groups. Of 178 implants, 48 (27%) lacked complete records and were excluded, and exclusion was more frequent for HR (33%) than for immediate implants (19%), although excluded implants resembled included ones in region, placement ISQ and ITV; this missingness could bias the results if it is related to stability, and available-case and worst-case analyses did not change the conclusions. The equivalence margin was chosen during the analysis and not prespecified, and equivalence was not tested for ITV. Correlation analyses were exploratory. The highest of four ISQ directions was recorded at placement and the lowest value at 3 months; the convention was applied identically in all groups, so it does not bias the comparison between protocols, but the two readings are differently defined, the magnitude of ISQ change (including the recovery of implants that started below 70) cannot be interpreted as a change in an identically defined measurement, and the directional values needed for a sensitivity analysis were not retained. The reliability of ISQ measurement was not assessed. Stability was measured at only two time points, so the early stability dip and the timing of recovery could not be described. The bone-level data lack a baseline and were measured once by one of two examiners, with reliability assessed only in a subsample. Sex-related differences in bone density could plausibly influence implant stability; however, sex was recorded but outcomes were not analyzed by sex, because the study was not designed or powered for sex-specific comparisons. Finally, implant survival could not be estimated: the analysis required a 3-month ISQ, and failure status was not ascertained over a defined follow-up period for all screened implants.

Prospective studies with a prespecified equivalence margin, standardized placement depth, stability measured at more time points and standardized baseline and follow-up radiographs would test these findings.

## 5. Conclusions

Within the limitations of this retrospective cohort, no difference in insertion torque, ISQ at placement or ISQ at 3 months was detected between the immediate protocol and delayed placement into grafted or healed ridges for subcrestal Morse-taper implants, and in the primary analysis the ISQ differences were statistically within a post hoc margin of ±5 units; at placement this was borderline in a sensitivity analysis, so the placement result is best read as no detected difference rather than demonstrated equivalence. The protocols differed in depth, grafting and healing management as well as timing, so these results describe the protocols and not timing alone. The relation between starting ISQ and ISQ gain is partly arithmetic; baseline-adjusted gain did not differ between protocols. Immediate implants with low placement stability reached ISQ 70 or more by 3 months when managed with the submerged or unloaded protocol used here. The suggestion that insertion torque tracks ISQ less closely in extraction sockets than in healed ridges is exploratory and should be tested prospectively.

## Figures and Tables

**Table 1:** Characteristics of the analyzed implants by placement group.

Characteristic	IM	DG	HR
Implants, *n*	56	30	44
Patients, *n* ^a^	40	26	32
Maxilla, *n* (%)	36 (64)	20 (67)	22 (50)
Maxillary anterior, *n* (%)	21 (38)	2 (7)	6 (14)
Maxillary posterior, *n* (%)	15 (27)	18 (60)	16 (36)
Mandibular anterior, *n* (%)	1 (2)	0 (0)	3 (7)
Mandibular posterior, *n* (%)	19 (34)	10 (33)	19 (43)
Diameter, mm, median (range) ^b^	4.5 (3.5–5.0)	4.5 (4.0–5.0)	4.25 (3.0–5.0)
Length, mm, median (range) ^b^	9 (8–15)	9 (8–11)	9 (8–13)
Diameter and length recorded, *n* (%)	36 (64)	24 (80)	28 (64)
ITV < 25 Ncm, *n* ^c^	7	5	11

IM, immediate placement; DG, delayed placement into a site grafted within the preceding 12 months; HR, delayed placement into a healed ridge; ITV, insertion torque value. ^a^ Patients could contribute implants to more than one group. ^b^ From the practice’s implant inventory log; available for 88 of 130 implants. ^c^ By protocol, immediate implants with ITV below 25 Ncm were submerged and those with 25 Ncm or more received a tissue former or provisional crown; delayed implants were submerged.

**Table 2:** Implant stability by placement group (mean ± SD; median (range).

Measure	IM (*n* = 56)	DG (*n* = 30)	HR (*n* = 44)	Delayed, DG + HR (*n* = 74)	P, 3 Groups ^a^	P, IM vs. Delayed ^a^
ITV, Ncm	31.2 ± 8.9; 33.5 (5–45)	30.9 ± 9.1; 32 (10–45)	29.7 ± 9.9; 35 (5–45)	30.2 ± 9.6; 32 (5–45)	0.59	0.42
ISQ at placement	72.3 ± 7.8; 75 (49–84)	74.1 ± 8.4; 76 (51–86)	71.4 ± 9.3; 73.5 (48–87)	72.5 ± 9.0; 74.5 (48–87)	0.70	0.76
ISQ at 3 months	78.8 ± 4.8; 79.5 (61–87)	79.7 ± 4.7; 80 (65–88)	79.2 ± 6.2; 80.5 (62–89)	79.4 ± 5.6; 80 (62–89)	0.85	0.57
ISQ change	+6.6 ± 7.4; 5.5 (−10–22)	+5.6 ± 7.6; 4.5 (−9–27)	+7.8 ± 7.3; 6.5 (−12–25)	+6.9 ± 7.5; 5.5 (−12–27)	0.55	0.57

^a^ Mixed-effects models with a random intercept per patient (likelihood-ratio test for three groups; Wald test for immediate vs. delayed). ITV, insertion torque value; ISQ, implant stability quotient.

**Table 3:** Between-group differences in stability from mixed-effects models (delayed minus immediate; random intercept per patient).

Outcome	Model	Difference (95% CI)	P	90% CI (TOST)	TOST P, ±5/±4/±3
ITV, Ncm ^a^	Unadjusted	−1.4 (−5.0 to 2.1)	0.42	—	—
	Region-adjusted	−0.8 (−4.5 to 2.9)	0.68	—	—
ISQ at placement	Unadjusted	−0.5 (−3.6 to 2.6)	0.76	−3.1 to 2.1	0.002/0.013/0.055
	Region-adjusted	−1.1 (−4.2 to 1.9)	0.47	−3.7 to 1.4	0.007/0.033/0.115
	Posterior sites only (*n* = 97)	−1.6 (−5.0 to 1.7)	0.34	−4.4 to 1.2	0.024/0.081/0.209
	Size- and region-adjusted (*n* = 88)	−1.8 (−5.6 to 2.0)	0.35	−5.0 to 1.4	0.050/0.129/0.269
ISQ at 3 months	Unadjusted	0.5 (−1.3 to 2.4)	0.57	−1.0 to 2.1	<0.001/<0.001/0.004
	Region-adjusted	0.4 (−1.4 to 2.2)	0.66	−1.1 to 1.9	<0.001/<0.001/0.002
	Posterior sites only (*n* = 97)	0.1 (−2.0 to 2.2)	0.92	−1.7 to 1.9	<0.001/<0.001/0.004
	Size- and region-adjusted (*n* = 88)	0.4 (−1.7 to 2.6)	0.70	−1.4 to 2.2	<0.001/<0.001/0.009
ISQ at 3 months, adjusted for placement ISQ	Unadjusted	0.6 (−1.0 to 2.2)	0.46	−0.8 to 2.0	<0.001/<0.001/0.002
	Region-adjusted	0.7 (−0.9 to 2.3)	0.40	−0.7 to 2.0	<0.001/<0.001/0.002

^a^ No equivalence margin was set for ITV and equivalence was not tested. ITV, insertion torque value; ISQ, implant stability quotient; CI, confidence interval; TOST, two one-sided tests (exact P for margins of ±5, ±4 and ±3 ISQ units; the ±5 margin was chosen post hoc).

**Table 4:** Spearman correlation between insertion torque and ISQ by placement group (95% CI from patient-level cluster bootstrap).

Group	*n*	ITV vs. Placement ISQ, ρ (95% CI)	ITV vs. 3-Month ISQ, ρ (95% CI)
IM	56	0.25 (−0.04 to 0.50)	0.07 (−0.22 to 0.36)
DG	30	0.22 (−0.12 to 0.54)	0.20 (−0.09 to 0.50)
HR	44	0.55 (0.25 to 0.78)	0.41 (0.12 to 0.68)
All	130	0.34 (0.16 to 0.50)	0.22 (0.07 to 0.37)

## Data Availability

The data presented in this study are available on request from the corresponding author due to patient privacy restrictions. The statistical analysis description and code are provided as Supplementary File S2. The de-identified implant-level dataset is available from the corresponding author on request.

## Supplementary Materials

The following supporting information can be downloaded at https://www.mdpi.com/article/10.3390/jcm15208184/s1, Table S1: STROBE checklist; File S2: description of the statistical analysis and analysis code (Python).

## Abbreviations

ASA, American Society of Anesthesiologists; CI, confidence interval; DG, delayed placement into a site grafted within the preceding 12 months; FDBA, freeze-dried bone allograft; HIPAA, Health Insurance Portability and Accountability Act; HR, delayed placement into a healed ridge; IM, immediate placement; ISQ, implant stability quotient; ITV, insertion torque value; PMMA, poly(methyl methacrylate); RFA, resonance frequency analysis; SD, standard deviation; TOST, two one-sided tests.
