# Supplementary material for: Primary and Secondary Stability of Subcrestal Morse-Taper Implants Placed Immediately into Extraction Sockets or into Grafted or Healed Ridges: A Retrospective Cohort Study
Source: J Clin Med. 2026 Oct 21;15(20):8184. doi: 10.3390/jcm15208184 (PMC13705761; doi:10.3390/jcm15208184)
Supplement: Supplementary file 1 [file jcm-15-08184-s001.zip › Supplementary_Table_S1_STROBE_checklist.pdf]

Supplementary Table S1. STROBE checklist (cohort study)

Manuscript: Primary and Secondary Stability of Subcrestal Morse-Taper Implants Placed Immediately into Extraction Sockets or into Grafted or Healed Ridges: A Retrospective Cohort Study

Section, table and figure numbers refer to the revised manuscript (jcm-4636135, second revision).

Item	No.	Recommendation	Reported in
**Title and abstract**	1	(a) Study design in title or abstract	Title (“Retrospective Cohort Study”); Abstract
		(b) Informative, balanced summary	Abstract
**Introduction**			
Background/rationale	2	Scientific background and rationale	Section 1, paragraphs 1–3
Objectives	3	Objectives, including prespecified hypotheses	Section 1, final paragraph (null hypothesis; secondary aims)
**Methods**			
Study design	4	Key elements of study design	Section 2.1
Setting	5	Setting, locations, relevant dates	Section 2.1 (private practice, Wausau, WI, USA; 2019–2022); Section 2.5
Participants	6	(a) Eligibility criteria, sources and methods of selection, follow-up	Section 2.2; Section 2.4 (12-week visit); Section 3.7 (failure status from practice records)
		(b) Matched studies	Not applicable

Item	No.	Recommendation	Reported in
Variables	7	Outcomes, exposures, predictors, confounders	Section 2.3 (exposure: IM/DG/HR); Section 2.4 (protocol differences in depth, flap, grafting and healing management, collinear with group); Section 2.5 (ITV, ISQ, ISQ change, bone level); Section 2.7 (region and implant size as covariates)
Data sources/measurement	8	Sources of data and methods of measurement	Section 2.5 (surgical motor; Penguin RFA, four directions: highest value recorded at placement, lowest at 3 months; radiographs; post hoc blinded re-read of 20 implants, ICC 0.93); Section 2.6
Bias	9	Efforts to address bias	Sections 2.6 and 2.7 (chart verification, complete-case rule, mixed models, region adjustment, sensitivity analyses); Section 3.1 (included vs. excluded implants); Section

Item	No.	Recommendation	Reported in
			4 (limitations)
Study size	10	How study size was arrived at	Section 2.2 (all eligible implants of a consecutive series); Section 3.1; Figure 1
Quantitative variables	11	Handling of quantitative variables	Section 2.5 (ISQ change; sign convention for bone level); Section 2.7 (ISQ < 70 threshold; ITV per 10 Ncm in interaction model); Section 3.4 and Figure 3 (ISQ bands; ITV < / ≥ 25 Ncm)
Statistical methods	12	(a) All statistical methods, including control for confounding	Section 2.7 (linear mixed-effects models with a random intercept per patient; region adjustment; TOST equivalence at ±5, ±4 and ±3 ISQ units)
		(b) Subgroups and interactions	Section 2.7 (three-group and pooled comparisons; ITV–ISQ correlations by group; ITV-by-group interaction model)
		(c) Missing data	Sections 2.2, 2.6 and 2.7 (complete-case analysis; ITV not imputed; available-case sensitivity analysis); Section

Item	No.	Recommendation	Reported in
			3.1 (included vs. excluded implants); Figure 1
		(d) Loss to follow-up	Section 3.6 (bone-level subset, 43 implants without follow-up film); Section 3.7 (failed implant; worst-case analysis)
		(e) Sensitivity analyses	Section 2.7 (posterior sites only; size- and region-adjusted; available-case; patient-level cluster bootstrap; baseline-adjusted ISQ); Section 3.3 and Table 3; Section 3.7 (worst-case imputation for the failed implant)
**Results**			
Participants	13	(a) Numbers at each stage	Section 3.1; Figure 1 (178 implants/111 patients screened; 130/85 analyzed; 87/59 with radiograph)
		(b) Reasons for non-participation	Section 3.1; Figure 1
		(c) Flow diagram	Figure 1
Descriptive data	14	(a) Characteristics of participants	Section 3.1; Table 1
		(b) Number with missing data per variable	Section 3.1 (implant size 88/130); Table 1; Section 3.6

Item	No.	Recommendation	Reported in
			(radiographs 87/130)
		(c) Follow-up time	Section 3.6 (median 10 months, range 3–41)
Outcome data	15	Numbers of outcome events or summary measures over time	Tables 2 and 4; Section 3.4; Section 3.7 (failure)
Main results	16	(a) Unadjusted and adjusted estimates with precision	Sections 3.2–3.3; Table 3 (unadjusted and adjusted mixed-model differences, 95% and 90% CI)
		(b) Category boundaries for continuous variables	Section 3.4 and Figure 3 (ISQ < 70, 70–76, ≥ 77; ITV < 25 Ncm)
		(c) Relative risk translation	Not applicable
Other analyses	17	Subgroups, interactions, sensitivity analyses	Sections 3.3–3.7; Tables 3 and 4
**Discussion**			
Key results	18	Summary with reference to objectives	Section 4, paragraph 1
Limitations	19	Limitations, sources of bias or imprecision	Section 4, limitations paragraph
Interpretation	20	Cautious overall interpretation	Section 4
Generalisability	21	External validity	Section 4, limitations paragraph (single surgeon, single

Item	No.	Recommendation	Reported in
			system)
**Other information**			
Funding	22	Funding source and role of funders	Funding; Conflicts of Interest
